# Unveiling the Impact of Smokers’ Self-Construals on the Effectiveness of Smoking Cessation Campaigns: A Comparative Analysis of E-Cigarettes and Combustible Cigarettes

**DOI:** 10.3389/ijph.2024.1606915

**Published:** 2024-05-23

**Authors:** Dong Hoo Kim, Ji Mi Hong

**Affiliations:** ^1^ Department of Advertising and Public Relations, College of Business and Economics, Chung-Ang University, Seoul, Republic of Korea; ^2^ Department of International Business and Trade, School of Global Convergence Studies, INHA University, Incheon, Republic of Korea

**Keywords:** smoking or vaping cessation campaigns, e-cigarettes, combustible cigarettes, selfconstruals, cigarette types

## Abstract

**Objective:**

This research conducted two studies in South Korea to explore the relationship between smokers’ self-construals and the types of cigarettes they use, emphasizing their combined effects on cessation campaign effectiveness.

**Methods:**

Study 1 explored how smokers’ self-construals influenced their intentions to quit smoking or vaping, considering their primary cigarette usage. Study 2 further investigated this relationship within cessation campaigns, employing messages framed by both self-construal (independent vs. interdependent) and cigarette type (combustible vs. electronic).

**Results:**

The results of Study 1 showed that individuals with a strong interdependent self-construal were more likely to express intentions to quit smoking or vaping when using e-cigarettes compared to combustible cigarettes. Similarly, Study 2 demonstrated that cessation messages for e-cigarettes were more effective in eliciting intentions to quit when presented with an interdependent self-construal frame, while messages for combustible cigarettes showed greater effectiveness with an independent self-construal frame.

**Conclusion:**

Campaigns solely focused on independent self-construals might not effectively persuade e-cigarette users to quit, as they may prioritize communal wellbeing over individual benefits. Adapting anti-e-cigarette campaigns to align with the values of interdependent self-construals could yield better outcomes in promoting cessation among e-cigarette users.

## Introduction

While global smoking rates have been declining since 1990 [1], the utilization of e-cigarettes has been rapidly increasing, reaching global sales of US$40 billion in 2023 [2]. This trend is particularly noticeable among teenagers, as e-cigarettes have become one of the most favored tobacco products among American youths since 2014 [3, 4], with approximately 13.1% of middle and high school students in the U.S. reporting vaping e-cigarettes in the past 30 days [5]. A similar pattern is observed in South Korea, where the Korea Disease Control and Prevention Agency (KDCPA) noted an increase in youth e-cigarette usage from 2.2% in 2017 to 3.2% in 2019 [6, 7].

The popularity of e-cigarettes among adolescents and young adults is largely attributed to their stylish designs, ease of use, and ability to be discreetly used in smoke-free areas [8]. Additionally, e-cigarette advertising often utilizes tactics appealing to youth, including promoting flavors, offering discounts, highlighting product design, featuring events like sports or bars, and incorporating emotional appeals and humor [9].

While e-cigarette advertising frequently portrays these products as safer alternatives to traditional combustible cigarettes [10–12], mounting evidence suggests that they can pose various health risks. E-cigarette use has been linked to adverse cardiovascular effects, respiratory symptoms, and compromised pulmonary immune function in adolescents [13–15]. Additionally, the nicotine found in e-cigarettes can lead to addiction and negatively affect adolescent brain development [16]. Furthermore, e-cigarettes can act as a gateway to smoking, encouraging the concurrent use of nicotine-containing and nicotine-free vapes [17], as well as traditional combustible cigarettes [18, 19].

The rising prevalence of e-cigarette use and growing concerns about associated health risks have spurred a demand for cessation campaigns aimed at curbing e-cigarette usage. England et al. [20] observed that adolescents harbored misconceptions about e-cigarettes, including beliefs that they were nicotine-free, harmless, and trendy. To address these misconceptions, they collaborated with adolescents, experts, and a marketing firm to develop the Rethink Vape campaign, focusing on three key messages: “What’s in the Vapor?,” “Health Risks,” and “Connections to Big Tobacco.” Results showed that teenagers exposed to the Rethink Vape materials experienced significant increases in vaping knowledge, perceived risk, individual vulnerability, and anti-vape intentions compared to those in the control group.

Liu and Yang [21] explored the combined impact of message format (narrative vs. non-narrative) and message framing (gain vs. loss) in e-cigarette prevention targeting young adults. They found that the gain-framed narrative reduced guilt compared to the gain-framed non-narrative, resulting in heightened risk perception and decreased intention to use e-cigarettes. Similarly, the loss-framed narrative evoked more sadness, leading to increased risk perception and reduced behavioral intention. These findings demonstrated that both transportation and discrete emotions served as mediators in the message’s impact on risk perception and behavioral intention.

Furthermore, Wang and Huang [22] examined the effects of narrative communication in correcting misinformation about e-cigarettes, focusing on message format (story vs. non-story) and message sidedness (one-sided vs. two-sided). Their findings indicated that the effectiveness of message format and sidedness varied depending on participants’ prior experience with e-cigarettes. Specifically, participants who had never used e-cigarettes favored the one-sided story; however, this preference declined among those with prior e-cigarette usage experience.

Despite efforts in previous studies, limitations persist wherein the message structures commonly employed in combustible smoking cessation contexts are merely transposed to e-cigarette use without accounting for the unique psychological profiles of e-cigarette users, including their self-concepts. Neglecting these nuances may result in ineffective health communication strategies that fail to resonate with e-cigarette users and overlook their specific needs and motivations for tobacco cessation.

In psychology, self-concepts encompass individuals’ beliefs, perceptions, and evaluations about themselves, including their personality traits, abilities, roles, and identities [23]. Self-construals, among various self-concepts, hold significant importance in health communication, representing the cultural and social frameworks that influence individuals’ perceptions of themselves in relation to others and their social environment, thus playing a pivotal role in shaping their health-related attitudes, beliefs, and behaviors [24–26].

According to Markus and Kitayama [27], self-construals are related to individuals’ beliefs regarding “the relationship between the self and others and, especially, the degree to which they see themselves as separate from others or as connected with others” (p. 226). They encompass two main dimensions: independent self-construal, which emphasizes autonomy, uniqueness, and individuality, and interdependent self-construal, which emphasizes interconnectedness, relationships, and social harmony [27, 28].

Cultures play a significant role in shaping individuals’ self-construals [27]. Western cultures typically emphasize independence, leading individuals in these cultures to frequently activate the independent self-construal and make it chronically accessible, whereas individuals in eastern cultures tend to prioritize interdependence [24]. Despite cultural influences on the accessibility of self-construals, it is believed that individuals can exhibit both independent and tjinterdependent self-construals regardless of their cultural backgrounds [29]. Similar to the concept of malleable self-concept proposed by Markus and Kunda [23], either self-construal can be more activated based on situational stimuli and primes [30, 31].

Individuals’ chronic and activated self-construal significantly influences their perception of themselves as smokers. For instance, research by Chang [32] indicates that smokers typically view themselves as more independent than non-smokers. When exposed to self-referential anti-smoking advertisements emphasizing their personal health risks from smoking, they tend to develop more negative attitudes toward smoking compared to messages highlighting the potential harm to their family. These findings prompt an exploration of how variations in self-construal might shape perceptions of e-cigarettes versus traditional combustible cigarettes.

Based on this research gap, this study suggests an interplay between self-construals and smoking types. Individuals with an independent self-construal, prioritizing personal autonomy and individualism, may view e-cigarettes as a means to assert control over their health choices. Conversely, those with an interdependent self-construal, valuing connections with others and social harmony, may consider the social implications of e-cigarette usage on their relationships and community health.

Specifically, given the significance of “less odor” and “reduced secondhand smoke effects” as primary advantages of e-cigarette usage among Korean smokers [12], individuals with an interdependent self-construal may find vaping particularly appealing. They are likely to be highly aware of the social impacts of vaping and may see e-cigarettes as a way to fulfill their social responsibilities and protect the wellbeing of those around them.

In contrast, the intense nicotine flavor in traditional combustible cigarettes could motivate smoking cessation efforts among individuals with an independent self-construal. Prioritizing personal health and wellbeing over social connections, these smokers may find the strong nicotine taste unpleasant, prompting them to reconsider their smoking habits to better align with their health goals.

To examine these hypotheses, we conducted two empirical studies analyzing individuals’ self-construals regarding two different types of cigarettes—e-cigarettes and combustible cigarettes. Study 1 investigated the variance in individuals’ self-construal based on the type of cigarette they used and its impact on their cessation intentions. Furthermore, Study 2 explored the effectiveness of campaigns by employing different self-construals in message framing as a strategy to promote smoking or vaping cessation for each type of cigarette.

## Methods

### Study 1

Study 1 aimed to investigate the relationship between smokers’ self-construals and the types of cigarettes they use concerning their intentions to quit smoking. Specifically, we hypothesized that e-cigarette smokers with a stronger interdependent self-construal would exhibit a greater intention to quit compared to those with an independent self-construal. Conversely, combustible cigarette smokers with a strong independent self-construal were expected to be more likely to quit smoking than those with an interdependent self-construal.

To test the proposed hypotheses, Study 1 conducted an online survey involving 125 Korean smokers recruited through Survey People, a research firm in South Korea. All participants received a briefing on the study’s objectives, provided consent, and received a monetary compensation of approximately $10 (₩15,000-KRW) for their participation. Each online survey took around 15–20 min per participant. Study 1 consisted predominantly of male participants (85%), while females accounted for 15%, consistent with the gender distribution of smoking prevalence in South Korea [32]. Regarding age distribution, participants were categorized as follows: 20 s (22%), 30 s (25%), 40 s (31%), and 50 s (22%).

Upon accessing the online survey, participants were screened to verify their smoker status, excluding nonsmokers based on specific criteria: smoking more than 5 packs (100 cigarettes) in their lifetime and smoking more than 1 cigarette in the past month, as defined by the World Health Organization. The online survey collected data on participants’ type of cigarette usage (e-cigarette or combustible cigarette), smoking habits (including daily consumption and duration), and perceptions of e-cigarettes (relevant only to e-cigarette smokers). Additional sample characteristics can be found in Supplementary Appendix SA.

Participants’ self-construals were then evaluated using the Singelis Self-Construal Scale [30], which was adapted to include 7 items and translated into Korean (*Cronbach’s α* = .82). Taking into account Korea’s strong collectivistic culture and its potential influence on individuals’ self-construals [33, 34], Study 1 focused exclusively on assessing participants’ interdependent self-construals and examining how their varying degrees may correlate with different types of smoking. For the precise phrasing of each scale item, see Supplementary Appendix SC.

Participants were also prompted to indicate their intention to quit smoking using the scale adapted from Wong and Cappella [35], serving as the dependent variable. This measure ranged from 1 (definitely will not) to 7 (definitely will), encompassing 5 items, such as “I will quit smoking completely and permanently in the next 3 months” (*Cronbach’s α* = .88). Finally, participants provided demographic information and were then debriefed and thanked for their participation.

## Results

To investigate the differences in smokers’ self-construals based on the types of cigarette they use and their impact on cessation intentions, we employed multiple regression analysis. Prior to conducting the regression analysis, a centered version of individuals’ self-construal was created by subtracting the mean scores (*M* = 4.53, *SD* = .73). Subsequently, a cross-product variable was generated to examine the interaction effect between smokers’ self-construals and cigarette types. The cessation intentions were then regressed onto individuals’ self-construals (centered), cigarette types (coded as 0 for combustible cigarettes and 1 for e-cigarettes), and the interaction term (self-construal * cigarette types).

As depicted in Table 1, the regression model yielded statistically significance (*R* ^*2*^ = .17, *F* [3,121] = 8.45, *p* = .00). Notably, the main effects of self-construals (*β* = .13, *t* = 1.12, *p* = .26) and cigarette types (*β* = .15, *t* = 1.7, *p* = .09) were not significant. However, a significant interaction effect was observed between smokers’ self-construals and cigarette types (*β* = .26, *t* = 2.21, *p* = .03).

**TABLE 1:** Multiple linear regression results for Study 1 (Unveiling the impact of smokers’ self-construals on the effectiveness of smoking cessation campaigns: A comparative analysis of E-cigarettes and combustible cigarettes, Republic of Korea, 2023).

	Cessation intention		
	*β*	SE	*t*
Smokers’ Self-Construals (Interdependent)	.35	.12	4.10^**^
*R* ^*2*^	.11		
Model F	16.83^**^		
Smokers’ Self-Construals (Interdependent)	.31	.12	3.63^**^
Cigarette Types	.14	.18	1.67
*R* ^*2*^		.14	
Model F		9.92^**^	
∆*R* ^*2*^		.02	
Incremental F		2.77	
Smokers’ Self-construals (Interdependent)	.13	.17	1.12
Cigarette Types	.15	.17	1.70
Smokers’ Self-Construals × Cigarette types	.26	.24	2.21^*^
*R* ^*2*^		.17	
Model F		8.45^**^	
∆*R* ^*2*^		0.03	
Incremental F		4.86^*^	

^*^ *p* < .05, ^**^ *p* < .01.

**FIGURE 1:**
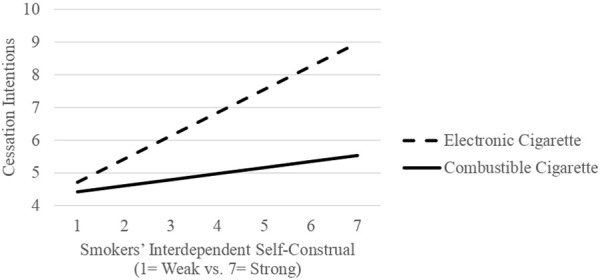
Study 1: The interaction between smokers’ self-construals and the types of cigarettes they use on cessation intentions (Unveiling the impact of smokers’ self-construals on the effectiveness of smoking cessation campaigns: a comparative analysis of E-cigarettes and combustible cigarettes, Republic of Korea, 2023).

Essentially, there was no statistical variation in the willingness of combustible cigarette smokers to quit based on their predominant self-construal. Conversely, among e-cigarette users, individuals with a stronger sense of interdependence demonstrated a greater intention to quit vaping (refer to Figure 1). These findings suggest that the association between e-cigarettes and interdependent self-construal may positively influence smokers’ intentions to quit smoking.

## Methods

### Study 2

Expanding on the findings of Study 1, Study 2 aimed to explore how cessation intentions were influenced by messages framed according to both cigarette types and self-construals within the context of anti-smoking and/or vaping campaigns. Using a between-subjects factorial design of 2 (cigarette type: combustible vs. electronic) × 2 (self-construal: independent vs. interdependent), we hypothesized a matching effect, expecting cessation messages for e-cigarettes to elicit stronger intentions to quit smoking when combined with an interdependent self-construal frame, while messages for combustible cigarettes would be more effective with an independent self-construal frame.

An online experimental survey was developed for Study 2, involving 125 Korean smokers recruited through the Survey People panel. After excluding two participants with incomplete responses, the analysis was conducted with a total of 123 participants (83% male, 17% female, average age = 39, *SD* = 10.39). Initially, participants were surveyed regarding their smoking status and habits to differentiate between users of combustible cigarettes and e-cigarettes (see Supplementary Appendix SB). Out of 123 respondents, 84 (68.3%) disclosed using electronic cigarettes, whereas 39 (31.7%) stated they did not. Based on their responses, participants who identified themselves as e-cigarette users were further questioned about their specific usage patterns, with three choices provided: a) solely using electronic cigarettes (*N* = 25), b) engaging in dual use but predominantly with electronic cigarettes (*N* = 30), and c) engaging in dual use but predominantly with combustible cigarettes (*N* = 29). Consequently, participants who exclusively used or predominantly used e-cigarettes were classified as e-cigarette users (*N* = 25 + 30 = 55, 45%), while those who exclusively used or predominantly used combustible cigarettes were categorized as combustible cigarette users (*N* = 39 + 29 = 68, 55%).

Following this, participants who categorized as e-cigarette users were randomly presented with one of two advertisements, each displaying the same e-cigarette image and wording but varying in terms of self-construal aspects (*N*_*electronic*__*x*__*independent*_ = 28 vs. *N*_*electronic*__*x*__*interdependent*_ *=* 27). Similarly, participants classified as users of combustible cigarettes were shown one of two advertisements, both displaying the same image and wording for combustible cigarettes but incorporating different self-construal aspects (*N*_*combustible x*_ _*independent*_ = 34 vs. *N*_*combustible x*_ _*interdependent*_ = 34).

Consistent with prior research [30], the self-construal aspects were manipulated using both textual messages and visual images in the stimuli. Under the independent self-construal condition, the messages primarily emphasized the potential health risks of smoking to the recipients themselves, featuring an image of a man smoking a cigarette alone against a white background. Conversely, the interdependent self-construal condition presented messages highlighting the potential dangers of smoking to loved ones, accompanied by an image of a man smoking a cigarette in the presence of his wife and child. All other factors remained constant except for the manipulation of these verbal and visual cues (see Supplementary Appendix SD). Following exposure to the advertisement, participants were asked to indicate their intention to quit smoking using the identical scale as in Study 1 [35] (*Cronbach’s α* = .89).

Furthermore, to gauge the effectiveness of the self-construal manipulation, participants were instructed to rate on a 7-point semantic differential scale with 3 items (adapted from Kareklas, et al. [34]; *Cronbach’s α* = .75). They were requested to specify if they perceived the advertisement as more pertinent to themselves or to others, such as “*the personal versus social consequences of smoking*,” “*the benefits of smoking cessation for themselves versus others*,” and “*the impact of smoking on personal versus others’ health*.”

Independent t-tests confirmed the successful manipulation of self-construal, revealing that participants in the interdependent self-construal condition (*M* = 4.69, *SD* = 1.14) were more inclined to associate the advertisement with thoughts about others rather than those in the independent self-construal condition (*M* = 3.95, *SD* = 1.29; *t* (121) = −3.40, *p* < .01). Finally, participants were asked about their demographic characteristics, and the survey duration averaged approximately 20–25 min.

## Results

To examine the hypotheses of Study 2, analysis of covariance (ANCOVA) was conducted, with smoking quantity and duration utilized as covariates in the analysis. The findings indicated that participants’ intentions to quit were significantly affected by the messages framed with varying cigarette types and self-construals [*F*(1, 117) = 5.59, *p* < .05, *η2* = .05] (see Table 2).

**TABLE 2:** Analysis of covariance results for Study 2 (Unveiling the impact of smokers’ self-construals on the effectiveness of smoking cessation campaigns: A comparative analysis of E-cigarettes and combustible cigarettes, Republic of Korea, 2023).

DV	Source	SS	df	MS	F
Cessation Intentions	Amount of Smoking	.041	1	.041	.034
	Period of Smoking	6.299	1	6.299	5.234*
	Cigarette Types	2.170	1	2.170	1.803
	Self-construals	1.028	1	1.028	.854
	Interaction	6.727	1	6.727	5.591*
	Error	140.784	117	1.203	
	Total	158.728	122		

^*^ *p* < .05, ^**^ *p* < .01.

**FIGURE 2:**
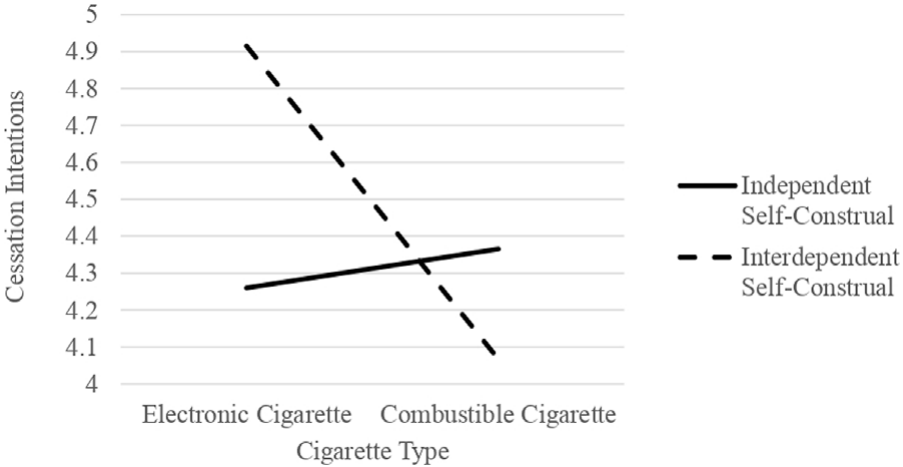
Study 2: The impact of cigarette types and self-construals on cessation intentions within the context of campaign messages (Unveiling the impact of smokers’ self-construals on the effectiveness of smoking cessation campaigns: a comparative analysis of E-cigarettes and combustible cigarettes, Republic of Korea, 2023).

A planned contrast showed that participants exposed to e-cigarette cessation messages expressed stronger intentions to quit [*F*(1, 117) = 4.88, *p* < .05, *η* ^*2*^ = .04] when the message incorporated the interdependent self-construal frame (*M* = 4.86, *SD* = 0.21) compared to the independent self-construal frame (*M* = 4.21, *SD* = 0.21). Conversely, those who viewed the combustible cigarette cessation message exhibited stronger intentions to quit when paired with the independent self-construal frame (*M* = 4.40, *SD* = 0.19) than the interdependent construal one (*M* = 4.12, *SD* = 0.19), although this variation did not reach statistical significance [*F*(1, 117) = 1.17, *p* > .05, *η* ^*2*^ = .01] (refer to Figure 2).

## Discussion

In two studies, we delve into the relationship between smokers’ self-construals and the types of cigarettes they used, focusing on how this connection influences their intentions to quit. The results from the two studies showed that neither smokers’ self-construal nor the type of cigarette influenced their intentions to quit smoking. This means that how smokers perceived themselves, either as independent entities or as part of social groups they belong to, has little to do with their willingness to quit smoking. It was also revealed that the type of cigarette they smoked was not solely indicative of their intention to quit smoking. Rather, when their self-construal was combined with the type of cigarette they smoked, a meaningful interaction occurred in their intention to quit smoking.

More specifically, Study 1 revealed that the intentions of combustible cigarette smokers to quit did not vary significantly based on their prominent self-construals. However, among e-cigarette users, those with a stronger sense of interdependence exhibited a greater intention to quit vaping. This indicates that cessation efforts aimed at e-cigarette users may yield better results when taking into account social contexts alongside individual factors.

Study 2 further explored this relationship within the context of cessation campaigns by framing messages based on two distinct types of cigarettes and self-construals. Despite a modest effect size (*η2* = .05), the interaction effect between cigarette types and self-construals in messages proved statistically significance. Specifically, e-cigarette cessation messages led to stronger intentions to quit when paired with the interdependent self-construal frame compared to those with an independent self-construal. Conversely, although not statistically significant, the trend was reversed for combustible cessation messages. This underscores the notion that individuals may possess different self-construals depending on the type of cigarettes they use, and messages reflecting this association can enhance the effectiveness of anti-smoking and/or vaping campaigns.

The present research makes significant theoretical contributions to the existing body of literature on self-construals and message framing within health communication. Our findings offer empirical evidence demonstrating how the choice of cigarette type (e-cigarette vs. combustible cigarette) can serve as a reflection of smokers’ self-construals (as evidenced in Study 1). Moreover, the results of Study 2 illustrate that cessation campaigns tailored to each type of cigarette can effectively enhance cessation intentions when they are aligned with congruent self-construals.

This finding aligns with prior research, which consistently indicates a preference for messages framed in accordance with individuals’ self-views [30, 36, 37]. Such congruent messaging fosters a “feel-right” experience [38, 39], thereby bolstering favorable attitudes and evaluations of the advocated message. By shedding light on the association between smokers’ self-construals and their cigarette usages, our research not only deepens our understanding of self-construal within health communication but also introduces a novel perspective for comprehending smoking and/or vaping behaviors.

Nevertheless, in both studies, our findings revealed a noteworthy matching effect, particularly evident in the context of e-cigarette conditions. This intriguing observation suggests that the distinctive characteristics of e-cigarettes, such as their reduced odor and minimized secondhand smoke effects, may influence users to perceive themselves as socially conscious individuals. Consequently, they may exhibit a more positive cessation intention and response to cessation messages framed within an interdependent self-construal context.

However, the situation with combustible cigarettes appears to be more complex. Unlike e-cigarettes, combustible cigarettes possess strong sensory attributes, including their potent nicotine flavor, lingering smell, and visible ash. These features could evoke a mix of emotions among smokers, potentially leading to conflicting self-construal activations. On one hand, smokers may experience feelings of guilt or concern over the harm their smoking habit inflicts on others, aligning with an interdependent self-construal. On the other hand, they may derive personal pleasure from the addictive taste and the sensory experience of smoking, reflecting elements of an independent self-construal.

This internal conflict may result in neither a clear activation of independent nor interdependent self-construal. To gain a deeper understanding of this phenomenon, future research should delve into the underlying mechanisms driving the observed differences between e-cigarettes and combustible cigarettes. Exploring factors such as sensory experiences, perceived health risks, and social perceptions surrounding each type of cigarette could provide valuable insights into this complex interplay.

Finally, the findings have significant implications for practitioners involved in anti-smoking and vaping campaigns. While many campaigns have traditionally been tailored to perceptions and strategies developed for traditional combustible cigarettes, it is crucial for campaign designers to recognize the unique factors associated with e-cigarettes. Smokers’ motivations and perceptions of e-cigarettes often differ substantially from those of combustible cigarettes, necessitating tailored approaches. Drawing from Markus and Kitayama’s recommendations (see Table 1, 1991, p. 230) [27], e-cigarette cessation campaigns might benefit from employing an interdependent self-construal framing, emphasizing external attributes, a sense of belonging, fulfilling responsibilities, and indirect communication. Conversely, general anti-smoking campaigns could incorporate an independent self-construal framing that emphasizes internal abilities, individual uniqueness, and direct communication. Thus, understanding the association between cigarette types and smokers’ self-construals provides valuable guidance for designing more effective cessation campaigns.

### Limitations and Future Research

Given Korea’s collectivistic cultural background, gender was not considered a primary or control variable in our study. This decision was informed by previous literature indicating that while studies conducted in the United States and similar individualistic nations suggest a prevalence of independent self-construal among males compared to females [40, 41], this gender disparity is not consistently observed in collectivist societies [42]. However, recognizing the potential impact of gender on self-construal, it could be a worthwhile factor to consider in future cross-cultural studies aimed at validating research outcomes.

In both studies, participants who reported using both e-cigarettes and combustible cigarettes were categorized based on their predominant usage pattern, either as e-cigarette users or combustible cigarette users. While efforts were made to account for dual usage in Study 2, it is crucial for future research to differentiate between dual users and exclusive users. By examining the unique characteristics and responses to cessation campaigns among these distinct groups, researchers can develop a more nuanced understanding of smoking behavior and effective intervention strategies.

Lastly, we delved into interdependent self-construal, particularly focusing on familial relationships such as those with family members and children. Existing literature underscores the distinction between relational-interdependent self-construal, which emphasizes the influence of specific, intimate relationships on one’s self-perception, and general interdependent self-construal, which extends this influence to encompass a broader network of social connections and communities [43]. This broader perspective suggests that variations in in-group and out-group dynamics may yield differing persuasive outcomes. Additionally, the concept of metapersonal self-construal has emerged, expanding upon individual and interpersonal self-construals by emphasizing interconnectedness with broader entities such as humanity, nature, or the universe [44, 45]. Consequently, it would be beneficial for future research to explore more nuanced dimensions of self-construal based on varying degrees of social ties and to examine their implications for the effectiveness of anti-smoking campaigns.

### Conclusion

Notwithstanding its limitations, this research contributes significantly to the growing body of literature and interventions related to e-cigarettes. It offers valuable insights that can guide researchers and practitioners in navigating the complex challenges associated with e-cigarette cessation campaigns. Notably, this research highlights the association between smokers’ self-construals and the types of cigarettes they use, emphasizing that tailoring campaigns to align with both cigarette types and self-construals can enhance cessation intentions. For instance, recognizing that e-cigarette smokers may prioritize concerns about the adverse effects of their smoking on others rather than themselves, anti-e-cigarette campaigns incorporating a strong interdependent self-construal (e.g., potential dangers of smoking to family) could prove more persuasive than those focusing solely on independent self-construals (e.g., personal health risks). This research underscores the significance of the relationship between smokers’ self-construal and cigarette types in determining the effectiveness of cessation campaigns.
