# Supplementary material for: Unveiling the Impact of Smokers’ Self-Construals on the Effectiveness of Smoking Cessation Campaigns: A Comparative Analysis of E-Cigarettes and Combustible Cigarettes
Source: Int J Public Health. 2024 May 23;69:1606915. doi: 10.3389/ijph.2024.1606915 (PMC11153756; doi:10.3389/ijph.2024.1606915)
Supplement: Supplementary file 1 [file DataSheet1.PDF]

Appendices

Appendix A: Sample Characteristics for Study 1 (*N* = 125)

			Electronic Cigarettes	Combustible Cigarettes	Total
Gender	Male	Count	58	48	106
		% of Total	46.4%	38.4%	84.8%
	Female	Count	6	13	19
		% of Total	4.8%	10.4%	15.2%
Age	20-29 years old	Count	16	11	27
		% of Total	12.8%	8.8%	21.6%
	30-39 years old	Count	12	19	31
		% of Total	9.6%	15.2%	24.8%
	40-49 years old	Count	18	21	39
		% of Total	14.4%	16.8%	31.2%
	50-60 years old	Count	18	10	28
		% of Total	14.4%	8.0%	22.4%
Smoking Period	5 years or less	Count	7	8	15
		% of Total	5.6%	6.4%	12.0%
	6-10 years	Count	12	12	24
		% of Total	9.6%	9.6%	19.2%
	11-15 years	Count	13	19	32
		% of Total	10.4%	15.2%	25.6%
	16-20 years	Count	8	8	16
		% of Total	6.4%	6.4%	12.8%
	21-25 years	Count	8	7	15
		% of Total	6.4%	5.6%	12.0%
	26-30 years	Count	4	4	8
		% of Total	3.2%	3.2%	6.4%
	31 years or more	Count	12	3	15
		% of Total	9.6%	2.4%	12.0%
Daily Smoking Amount	Less than 10 cigarettes	Count	31	26	57
		% of Total	24.8%	20.8%	45.6%
	11-20 cigarettes	Count	24	25	49
		% of Total	19.2%	20.0%	39.2%
	21-30 cigarettes	Count	8	7	15
		% of Total	6.4%	5.6%	12.0%
	Exceeding 30 cigarettes	Count	1	3	4
		% of Total	0.8%	2.4%	3.2%
Number of Past Attempts to Quit Smoking	0 times	Count	7	5	12
		% of Total	5.6%	4.0%	9.6%
	1 times	Count	14	9	23
		% of Total	11.2%	7.2%	18.4%
	2 times	Count	19	20	39
		% of Total	15.2%	16.0%	31.2%
	3 times	Count	9	17	26
		% of Total	7.2%	13.6%	20.8%

	4 times	Count	1	2	3
		% of Total	0.8%	1.6%	2.4%
	5 times	Count	1	1	2
		% of Total	0.8%	0.8%	1.6%
	More than 6 times	Count	13	7	20
		% of Total	10.4%	5.6%	16.0%
Total		Count	64	61	125
		% of Total	51.2%	48.8%	100.0%

Appendix B: Sample Characteristics for Study 2 (*N* = 123)

			Electronic Cigarettes	Combustible Cigarettes	Total
Gender	Male	Count	42	60	102
		% of Total	34.1%	48.8%	82.9%
	Female	Count	13	8	21
		% of Total	10.6%	6.5%	17.1%
Age	20-29 years old	Count	15	19	34
		% of Total	12.2%	15.4%	27.6%
	30-39 years old	Count	19	14	33
		% of Total	15.4%	11.4%	26.8%
	40-49 years old	Count	9	16	25
		% of Total	7.3%	13.0%	20.3%
	50-60 years old	Count	12	19	31
		% of Total	9.8%	15.4%	25.2%
Smoking Period	10 years or less	Count	25	30	55
		% of Total	20.3%	24.4%	44.7%
	11-20 years	Count	17	14	31
		% of Total	13.8%	11.4%	25.2%
	21-30 years	Count	9	15	24
		% of Total	7.3%	12.2%	19.5%
	31-40 years	Count	4	8	12
		% of Total	3.3%	6.5%	9.8%
	41 years or more	Count	0	1	1
		% of Total	0.0%	0.8%	0.8%
Daily Smoking Amount	Less than 10 cigarettes	Count	30	24	54
		% of Total	24.4%	19.5%	43.9%
	11-20 cigarettes	Count	20	32	52
		% of Total	16.3%	26.0%	42.3%
	21-30 cigarettes	Count	5	9	14
		% of Total	4.1%	7.3%	11.4%
	Exceeding 30 cigarettes	Count	0	3	3
		% of Total	0.0%	2.4%	2.4%
Number of Past Attempts to Quit Smoking	0 times	Count	4	9	13
		% of Total	3.3%	7.3%	10.6%
	1 times	Count	11	11	22
		% of Total	8.9%	8.9%	17.9%
	2 times	Count	17	25	42

		% of Total	13.8%	20.3%	34.1%
	3 times	Count	8	13	21
		% of Total	6.5%	10.6%	17.1%
	4 times	Count	3	3	6
		% of Total	2.4%	2.4%	4.9%
	5 times	Count	2	1	3
		% of Total	1.6%	0.8%	2.4%
	More than 6 times	Count	10	6	16
		% of Total	8.1%	4.9%	13.0%
Total		Count	55	68	123
		% of Total	44.7%	55.3%	100.0%

Appendix C: Measures

General Smoking Behaviors

1. Have you smoked more than 5 packs (100 cigarettes) in your lifetime or more than 1 cigarette in the past month?
   a) Yes b) No
2. When did you start smoking?
   a) Under 20 years old
   b) 20-29 years old
   c) Over 30 years old
3. How long have you been smoking?
   a) 10 years or less
   b) 11-20 years
   c) 21-30 years
   d) 31-40 years
   e) 41 years or more
4. How many cigarettes do you smoke per day?
   a) Less than 10 cigarettes
   b) 11-20 cigarettes
   c) 21-30 cigarettes
   d) Exceeding 30 cigarettes
5. How many times have you tried to quit smoking in the past?
   a) 0 times
   b) 1 time
   c) 2 times
   d) 3 times
   e) 4 times
   f) 5 times
   g) More than 6 times
6. Why did you try to quit smoking?
   a) To maintain fitness

8. If you use electronic cigarettes, what is your current smoking habit?
   a) Solely using electronic cigarettes.
   b) Engaging in dual use but primarily with electronic cigarettes.
   c) Engaging in dual use but predominantly with combustible cigarettes.
9. How long have you been using electronic cigarettes?
   a) Less than 6 months
   b) More than 6 months to less than 1 year
   c) More than 1 year to less than 3 years
   d) 3 years or more
10. Why do you use electronic cigarettes?
   a) It will help me quit smoking.
   b) It is less harmful to health than combustible cigarettes.
   c) To reduce the amount of combustible cigarette smoking.
   d) It smells less than combustible cigarettes.
   e) The taste and scent of electronic cigarettes are better than combustible cigarettes.
   f) Because there are no restrictions on location to use electronic cigarettes.
   g) Others (please specify)
11. What are your thoughts on electronic

b) Health deterioration
c) Family recommendation
d) Lack of designated smoking areas
e) To enhance personal image
f) Co-worker pressure
g) Other (please specify)

7. Do you use electronic cigarettes?
a) Yes b) No

cigarettes?
a) Supplementary to combustible cigarettes
b) Replacement for combustible cigarettes
c) Novel type of cigarettes
d) Aid in quitting smoking
e) Other (please specify)

Self-Construals (Singelis, 1994)

1. It is important to me respect decisions by the group.
2. It is important to me to maintain harmony within my group.
3. I will sacrifice my self-interest for the benefit of the group I am in.
4. I will stay in a group if they need me, even when I'm not happy with the group.
5. I often have the feeling that my relationships with others are more important than my own accomplishments.
6. I have respect to the authority figures with whom interact.
7. My happiness depends on the happiness of those around me.

Smoking Cessation Intentions (Wong & Cappella, 2009)

''How likely is it that in the next 3 months you will ________''

1. quit smoking completely and permanently.
2. reduce the number of cigarettes you smoke in a day.
3. talk to someone (friend, family member, spouse) about quitting smoking.
4. seek counseling/support to help you quit smoking.
5. enroll in a smoking cessation program if one were available to you at minimal cost and easy access.

Appendix D: Advertising Stimuli for Study 2

Combustible Cigarette × Independent Self-Construal

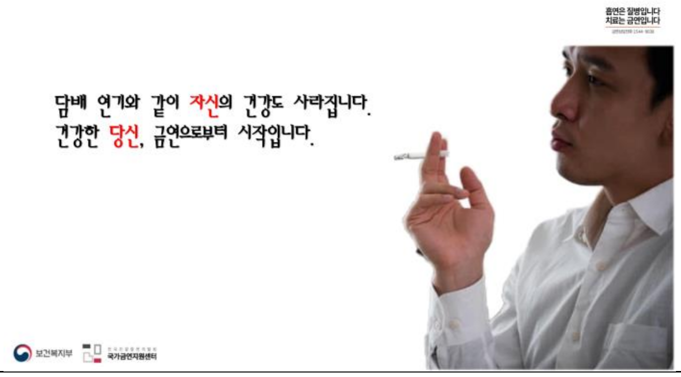

Electronic Cigarette × Interdependent Self-Construal	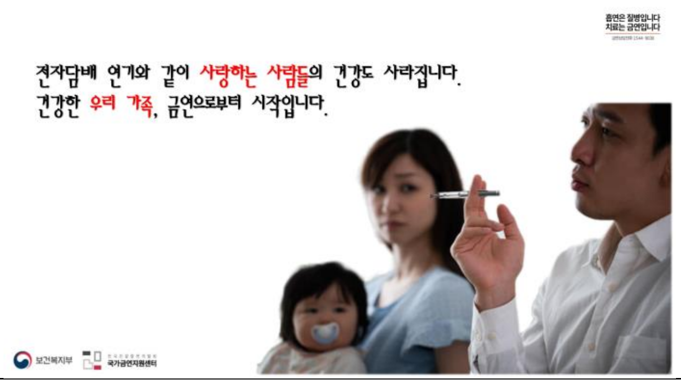
